# Functional Interrogation of an Odorant Receptor Locus Reveals Multiple Axes of Transcriptional Regulation

**DOI:** 10.1371/journal.pbio.1001568

**Published:** 2013-05-21

**Authors:** Alexander Fleischmann, Ishmail Abdus-Saboor, Atef Sayed, Benjamin Shykind

**Affiliations:** 1 Center for Interdisciplinary Research in Biology, College de France, Paris, France; 2 Weill Cornell Medical College in Qatar, Qatar Foundation–Education City, Doha, Qatar; North Carolina State University, United States of America

## Abstract

The odorant receptor (OR) genes constitute the largest mammalian gene family and are expressed in a monogenic and monoallelic fashion, through an unknown mechanism that likely exploits positive and negative regulation. We devised a genetic strategy in mice to examine OR selection by determining the transcriptional activity of an exogenous promoter homologously integrated into an OR locus. Using the tetracycline-dependent transactivator responsive promoter (tet_o_), we observed that the OR locus imposes spatial and temporal constraints on tet_o_-driven transcription. Conditional expression experiments reveal a developmental change in the permissiveness of the locus. Further, expression of an OR transgene that suppresses endogenous ORs similarly represses the OR-integrated tet_o_. Neurons homozygous for the tet_o_-modified allele demonstrate predominantly monoallelic expression, despite their potential to express both copies. These data reveal multiple axes of regulation, and support a model of initiation of OR choice limited by nonpermissive chromatin and maintained by repression of nonselected alleles.

## Abstract 2

A transgenic approach in mice allows the functional interrogation of an odorant receptor locus *in vivo* and reveals characteristics of its monogenic and monoallelic expression.

## Author Summary

Odorant receptor (OR) gene choice is a paradigmatic example of transcriptional regulation in which each olfactory sensory neuron selects a single OR from a repertoire of over 1,000 genes. Two mechanistic models of OR choice have been proposed. One postulates the existence of a specialized transcriptional machinery that selects just one OR allele, while a second, kinetic model proposes that OR chromatin is intrinsically nonpermissive, such that inefficient activation during a critical window of time restricts expression to a single OR allele. Here, we used a transgenic approach in mice in which we inserted a conditionally regulated exogenous promoter into an OR locus by homologous recombination in embryonic stem cells. The resulting novel mouse lines allowed the functional interrogation of the OR locus in vivo during development of the olfactory epithelium, enabling us to directly test models of OR choice. Using this experimental strategy we found that OR loci are indeed slow to activate and that the subsequent phenomenon of spatial restriction of OR expression is accomplished by repression. We also observed a developmental shutdown of OR loci concomitant with expression of the OR repertoire. Together, these experiments provide prima facie evidence for a kinetic model of initiation of OR gene choice, coupled with repression of nonselected OR alleles.

## Introduction

Olfactory sensory neurons are activated by odors in the periphery and transmit neural signals centrally to produce the perception of smell. On a molecular level, the diversity of odorous molecules is accommodated by a large number of G-protein-coupled odorant receptors (ORs), which form the largest gene family in mammals [1]. In rodents, individual olfactory sensory neurons select a single OR from more than 1,300 encoded in the genome [2]–[4], and choose one allele at random from which to transcribe it [5]. Neurons expressing the same OR are found scattered in broad zones that stretch across the olfactory epithelium [6],[7] and project their axons to a pair of discrete loci in the olfactory bulb, forming glomeruli at stereotypical positions [8]–[10]. Activation by odor results in a sparse pattern of activity in the olfactory bulb [11]–[13]. In this way a map is formed in the olfactory bulb in which odor identity may be encoded by unique patterns of glomerular activity. The OR molecules themselves play a prominent role in the positioning of the glomeruli, with subtle changes in the amino acid sequence of the ORs altering their glomerular location [10]. The biological rationale for the extreme transcriptional selectivity of OR regulation may in part be to take advantage of the sensitivity of the system to OR sequence heterogeneity: greater neuronal diversity allows greater olfactory discrimination. Thus, the OR selection process generates on the order of 2,500 different sensory neurons and is a critical first step in the generation of the olfactory circuit from the nose to the brain.

The process of olfactory receptor choice may be conceptually divided into two phases: an initiation stage, followed by a maintenance period, in which the expression of a single OR gene is preserved for the life of the neuron [14]. It is critical that the selected OR be the stable choice of the neuron, as a change in receptor would alter the ligand sensitivity of the neuron and confound the sensory map in the bulb. Several groups have examined the stability of receptor choice and found that expression of an OR gene is maintained by a feedback signal elicited by functional receptor [15]–[17]. The effect of the feedback on OR choice is thought to involve either the stabilization of a unique transcriptional machinery on the selected OR allele, or the prevention of activation of additional ORs by suppression [17]–[19]. Evidence for suppression has emerged from experiments with transgenes in which the OR coding region was suggested to be the cis-acting substrate for feedback repression [19]. It is possible that elements of both models function during the feedback process.

The mechanism of initiation of OR choice is less well understood but has been proposed to involve a process that limits expression to only a single allele at a time. In one model a unique transcriptional apparatus or transcriptional factory [20] has been suggested to activate just one OR allele at a time [17],[21],[22]. Recent DNA fluorescent in situ hybridization experiments have demonstrated that OR genes are clustered in discrete loci surrounding pericentromeric heterochromatin [23]. Intriguingly, a single allele of a unique genomic region on Chromosome 14 harboring a locus-control-region-like sequence termed H [16],[24],[25] was found to co-localize in trans in the nucleus with the one expressed OR [22]. This finding provided an attractive candidate for such a singular selection machinery. However, the functional significance of this co-localization remains unclear, as knock-out studies have found that H is only able to function in cis [26]. In a different model of initiation, a kinetic mechanism is invoked to limit the initial activation of OR alleles to one [14],[17],[27]. This model proposes that receptor genes share regulatory elements but that OR gene transcription is initially so inefficient that only one allele is likely to be activated during a given window of time. Indeed, recent studies have demonstrated that OR genes bear the hallmarks of repressed chromatin [27]. In either model, the successful expression of an OR leads to a feedback mechanism that halts the process and maintains the expression of a solitary member of the OR repertoire.

In the kinetic model of OR choice, repressive receptor gene chromatin may be invoked to slow the activation process. However, if a singular apparatus does choose receptor genes, OR chromatin may need to be permissive to allow access to the machinery. In the maintenance phase of OR regulation, the feedback signal could initiate the formation of OR heterochromatin and prevent the activation of additional receptors in a cell. Thus, an assessment of the functional state of an endogenous OR locus at different stages during the expression of the OR repertoire in the olfactory epithelium would further our understanding of the mechanisms involved in this gene regulatory process.

We devised a genetic strategy to examine the functional state of an endogenous OR gene in vivo by examining its permissiveness to transcription. In this approach we inserted the tetracycline-dependent transactivator responsive promoter (tet_o_) [28], at the transcriptional start site of the P2 OR gene [21], by homologous gene targeting in mouse embryonic stem cells (ES cells), to make a series of alleles subject to conditional activation. With these modified P2 alleles we may functionally “interrogate” the OR locus in vivo by attempting to activate its transcription with the tetracycline-dependent transactivator (tTa) [28]. As all of the flanking P2 sequences sufficient for regulation are preserved in these minimally modified alleles (unpublished data), we anticipate that regulatory constraints imposed upon the endogenous OR promoter will similarly impinge upon the exogenous tet operator. Further, this strategy lets us take advantage of the conditional activation of the tTa system to probe temporal changes in OR chromatin, by staged administration of doxycycline [28],[29].

Using this approach we have revealed important parameters of OR gene regulation. It is possible to activate the OR from within its locus, and we observe zonal regulation of the tet_o_, suggesting that this hallmark of OR gene expression is accomplished by repression. Within the P2 zone, the tet-modified allele is sparsely expressed in young mice but slowly increases in frequency over time. Remarkably, pre-activation of these alleles with tTa results in a stable, tTa-independent over-expression. Using staged administration of doxycycline to regulate the activation of the tet-modified alleles, we observe a developmental change in permissiveness that is concurrent with the maturation of the epithelium and is not dependent on the presence of the coding region of the receptor. Despite the continuous presence of tTa, the tet-operator-linked P2 gene is suppressed by the pervasive expression of an OR transgene previously demonstrated to repress the endogenous repertoire [18], an effect that is independent of the OR open reading frame. Finally, in mice homozygous for the tet-modified alleles, the tTa-driven expression of the OR is observed to be largely monoallelic, despite the genetic potential for biallelic activation, suggesting the existence of a functional asymmetry in the OR alleles.

Together these experiments lend support for a kinetic model of OR choice, governed by limited initial activation and maintained by the feedback repression of nonselected receptor genes.

## Results

### Generation of tet-P2 Alleles

**Figure 1:**
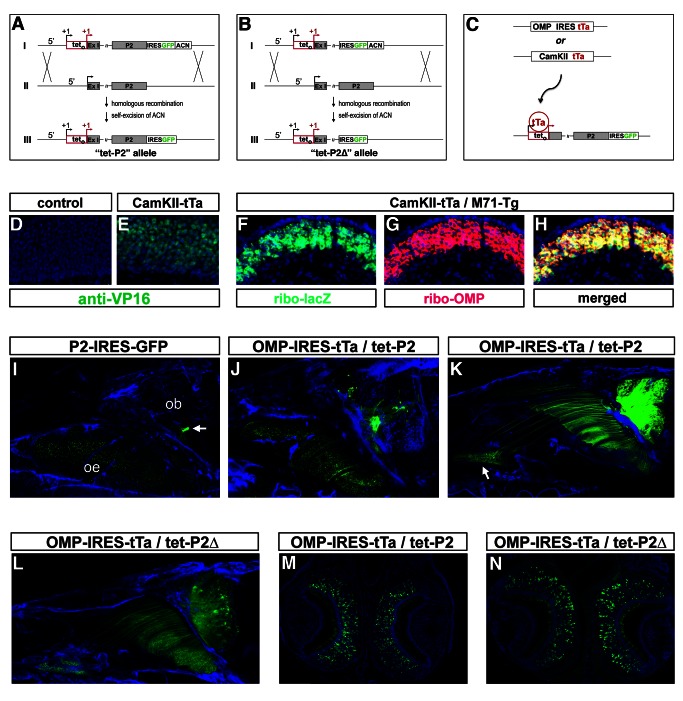
Construction of the tet-P2 and tet-P2Δ alleles and their expression in the olfactory sensory epithelium. (A) Modification of the endogenous P2 locus by homologous recombination to generate the tet-P2 allele. (I) The tet-P2-targeting construct allows bicistronic expression of the P2 OR protein and the marker protein GFP, both driven by the tet operator inserted at the start site of transcription of the P2 locus. Flanking P2 promoter regions are preserved in the construct, shifted 5′ of the tet operator. (II) The unmodified genomic P2 locus. (III) Homologous recombination in mouse ES cells followed by self-excision of the ACN selection cassette yields the tet-P2 allele. (B) Modification of the endogenous P2 locus by homologous recombination to generate the tet-P2Δ allele. (I) The tet-P2Δ-targeting construct allows expression of the marker protein GFP driven by the tet operator, inserted at the start site of transcription of the P2 locus, in the absence of the P2 coding region. Flanking P2 promoter regions are preserved in the construct, shifted 5′ of the tet operator. (II) The unmodified genomic P2 locus. (III) Homologous recombination in mouse ES cells followed by self-excision of the ACN selection cassette yields the tet-P2Δ allele. (C) Diagram of the genetic strategy used to examine the permissiveness of the tet_o_-modified P2 alleles in the mouse olfactory epithelium in vivo. The tet-P2 and tet-P2Δ alleles have the potential to be transcribed in all olfactory sensory neurons of the olfactory epithelium by the ubiquitous expression of tTa from either the modified OMP locus or the CaMKII-tTa transgene. (D and E) Ubiquitous expression of tTa from the CaMKII transgene demonstrated by immunohistochemical detection of the tTa protein by anti-VP16 antiserum. (D) Absence of tTa IHC signal in negative control tissue and (E) pervasive expression of tTa (green) in coronal sections of olfactory epithelium of CaMKII-tTa animals. Nuclei (blue) are counterstained with Toto-3. (F–H) Pervasive expression of a tet_o_-controlled OR (M71) transgene by the CaMKII-tTa transgene, as shown by RNA in situ hybridization. (F) Signal generated by riboprobes directed against mRNA synthesized from the M71 transgene (green); (G) riboprobes directed against OMP mRNA (red); (H) merged in situ signals. Nuclei (blue) are counterstained with Toto-3. (I–L) Endogenous GFP fluorescence in whole-mount preparations (parasagittal) of mouse olfactory epithelia reveals expression of control and tet_o_-modified P2 alleles. (I) Expression of the P2 allele revealed by GFP fluorescence (green) in control P2-IRES-GFP animals at P14 in sensory neurons of the olfactory epithelium (oe) and the projection of their GFP-positive axons into the olfactory bulb (ob). The GFP-positive P2 glomerulus is indicated (arrow). Nuclei (blue) are counterstained with Toto-3. (J) Expression of the tet-P2 allele revealed by GFP fluorescence (green) in OMP-IRES-tTa/tet-P2 animals at P14. GFP fluorescence also reveals multiple glomeruli in the olfactory bulb. Nuclei are revealed by Toto-3 counterstain (blue). (K) Expression of the tet-P2 allele revealed by GFP fluorescence (green) in OMP-IRES-tTa/tet-P2 animals at P60. Expression of tet-P2 in the VNO is indicated (arrow). GFP-positive fibers pervasively innervate the olfactory bulb. Nuclei are revealed by Toto-3 counterstain (blue). (L) Expression of the tet-P2Δ allele in the olfactory epithelium and VNO revealed by GFP fluorescence (green) in OMP-IRES-tTa/tet-P2Δ animals at P60. GFP-positive fibers pervasively innervate the olfactory bulb. Nuclei are revealed by Toto-3 counterstain (blue). (M and N) Expression of the tet_o_-modified P2 alleles in the VNO. (M) Coronal section through the VNO reveals extensive expression of the tet-P2 allele in OMP-IRES-tTa/tet-P2 animals at P60. Nuclei (blue) are counterstained with Toto-3. (N) Coronal section through the VNO reveals widespread expression of the tet-P2Δ allele in OMP-IRES-tTa/tet-P2Δ animals at P60. Nuclei (blue) are counterstained with Toto-3.

We used a gene targeting approach to examine the transcriptional permissiveness of a mouse OR gene, P2, in its chromosomal locus in vivo. In this strategy we inserted tet_o_ into the 5′ region of the endogenous P2 gene, to allow tTa [28] to functionally “interrogate” the locus by attempting to drive transcription across the P2 gene (Figure 1A–1C). Using homologous recombination in mouse ES cells, we generated a genetically modified mouse line (tet-P2) in which the tet_o_ was inserted at the start site of transcription of the P2 gene [21], while retaining the 5′ upstream regions required for endogenous P2 expression (unpublished data; Figure 1A). The green fluorescent marker protein GFP, linked to an internal ribosomal entry site (IRES), was inserted into the 3′ noncoding region of the P2 gene [30] to monitor its transcriptional activation (Figure 1A). Thus, all neurons that express the tet-P2 allele would synthesize a bicistronic mRNA allowing the translation of both the P2 receptor and GFP. To examine the role of the OR coding region in the initiation or maintenance of singular OR choice, we also generated tet-P2Δ, a mouse line bearing a modification of the P2 locus analogous to the tet-P2 allele, except for the deletion of the P2 coding region (Figure 1B).

To accurately assess the transcriptional permissiveness of the tet_o_-bearing P2 alleles, it is critical that tTa be pervasively expressed across the olfactory neuroepithelium. We therefore used two mouse lines that express tTa in olfactory sensory neurons, as shown in Figure 1C: OMP-IRES-tTa and CaMKII-tTa [29],[31]. The olfactory marker protein (OMP) is expressed in all mature olfactory sensory neurons [32]. Correspondingly, the OMP-IRES-tTa line co-expresses OMP and tTa in mature olfactory sensory neurons through an IRES element linked to the tTa gene and inserted into the 3′ UTR of OMP [31]. The CaMKII-tTa strain bears a transgenic construct in which the production of tTa is directed by the CaMKII gene promoter [29]. To determine the frequency of expression of tTa in the olfactory epithelium of CaMKII-tTa mice, we examined tissue by immunohistochemistry (IHC) with antiserum directed against the VP16-derived activation domain of the tTa protein. We observed pervasive expression of tTa in olfactory sensory neurons (>95%) of CaMKII-tTa mice, compared to controls (Figure 1D versus 1E, and data not shown). To confirm that CaMKII-tTa mice can indeed activate tet_o_-linked genes in the olfactory epithelium we crossed the CaMKII-tTa line with a previously generated strain (M71-tg) that harbors a transgenic construct in which the OR M71 and the marker protein tau-lacZ are under the control of the tet operator; this transgene can be activated in the vast majority of olfactory sensory neurons by OMP-IRES-tTa [18]. We verified that CaMKII-tTa similarly drives expression of the M71-tg in >95% of the olfactory neurons (Figure 1F–1H), confirming that CaMKII-tTa provides high-frequency expression of tTa. Importantly, as both the P2 and OMP genes reside on Chromosome 7 [21], it is not feasible to generate mice bearing both OMP-IRES-tTa and homozygous modification of the P2 alleles. To circumvent this problem we used the CaMKII-tTa line to allow the generation and analysis of homozygous tet-P2 animals.

### Expression of tet-P2 Alleles

In initial experiments we crossed mice carrying the tet-P2 or tet-P2Δ allele with the OMP-IRES-tTa line, and the resulting compound heterozygous animals were analyzed for GFP expression in whole-mount preparations. Activation of the tet-P2 allele by tTa results in sparse expression across the olfactory epithelium at P14, as observed by whole-mount fluorescent microscopy (Figure 1J). In comparison to control animals bearing a P2-IRES-GFP allele [30], in which GFP is expressed from the P2 locus under the control of the endogenous promoter, tTa elicits only a ∼5-fold increase in the frequency of expression of the tet-P2 allele (from 0.1% to 0.5% of the cells) in OMP-IRES-tTa/tet-P2 animals (Figure 1I and 1J, and data not shown). In the control P2-IRES-GFP animals, GFP^+^ axons project to the olfactory bulb and form a glomerulus at a stereotypical position [10]. Axons from tet-P2-expressing neurons in P14 OMP-IRES-tTa/tet-P2 animals form multiple glomeruli, clustered in a region of the bulb corresponding to that of the wild-type P2 glomerulus (Figure 1I, arrow), as well as scattered at ectopic positions (Figure 1J). In adult (>P60) OMP-IRES-tTa/tet-P2 animals, tTa-driven tet-P2 transcription becomes more pervasive, with expression seen in the main olfactory epithelium and in the vomeronasal organ (VNO) (Figure 1K, arrow) and a concomitant increase in the extent of innervation of the olfactory bulb (Figure 1K). Similar expression is observed when OMP-IRES-tTa drives activation of the deletion allele tet-P2Δ, with expression in the main olfactory epithelium and VNO and broad innervation of the olfactory bulb (Figure 1L). The overall frequency of tTa-driven expression of the tet-P2 and tet-P2Δ alleles in the olfactory epithelium in adult animals was determined by immunohistochemical analyses of neurons dissociated from the olfactory epithelium. We observed in bulk-dissociated cells from the olfactory epithelium that 55% of all OMP^+^ cells (*n* = 629) express the tet-P2 allele and 47% express the tet-P2Δ deletion allele (*n* = 452) in adult mice (data not shown). We observed comparable frequencies of expression of the tet_o_-driven allele in coronal sections through the VNOs of OMP-IRES-tTa/tet-P2 and OMP-IRES-tTa/tet-P2Δ adult animals (Figure 1M and 1N).

### Expression of tet-P2 Alleles Is Zonal

**Figure 2:**
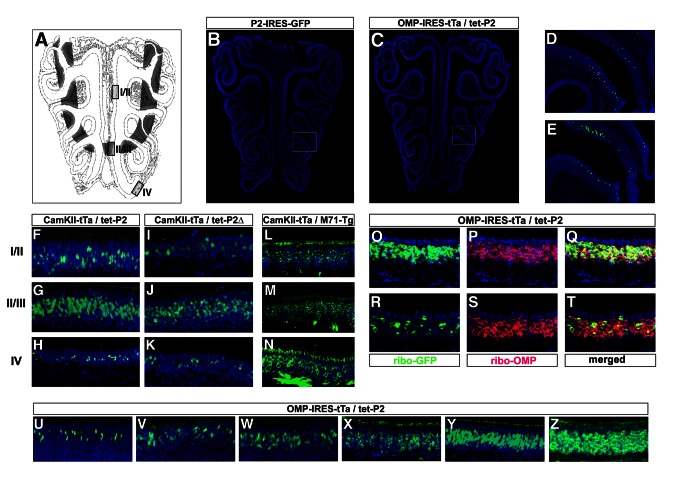
Frequency and zonal restriction of tTa-driven tet_o_-modified P2 alleles in the olfactory epithelium. (A) Diagram of the olfactory epithelium showing zones of OR expression. The shaded region is the II/III zone of P2 expression. Areas in black boxes depict regions shown in (F–N). (B) Coronal section through the olfactory epithelium of a P2-IRES-GFP control animal reveals expression of the P2 allele at P14. Sections were subject to anti-GFP IHC (green), and nuclei were counterstained with Toto-3 (blue). (C) Coronal section through the olfactory epithelium of a OMP-IRES-tTa/tet-P2-IRES-GFP animal reveals expression of the tet-P2 allele driven by tTa at P14. Sections were subject to anti-GFP IHC (green), and nuclei were counterstained with Toto-3 (blue). (D) High-power image of the boxed region in (B). (E) High-power image of the boxed region in (C). (F–H) Coronal sections through the olfactory epithelium of a CaMKII-tTa/tet-P2-IRES-GFP animal reveal the zonal restriction of expression of the tet-P2 allele driven by tTa at P75 in zone I/II (F), zone II/III (G), and zone IV (H). Sections were subject to anti-GFP IHC (green), and nuclei were counterstained with Toto-3 (blue). (I–K) Coronal sections through the olfactory epithelium of a CaMKII-tTa/tet-P2Δ-IRES-GFP animal reveal the zonal restriction of expression of the tet-P2 allele driven by tTa at P75 in zone I/II (I), zone II/III (J), and zone IV (K). Sections were subject to anti-GFP IHC (green), and nuclei were counterstained with Toto-3 (blue). (L–N) Coronal sections through the olfactory epithelium of a CaMKII-tTa/M71-Tg animal show pervasive expression of tet-linked M71 transgene driven by tTa at P60 in zone I/II (L), zone II/III (M), and zone IV (N). Sections were subject to anti-lacZ IHC (green), and nuclei were counterstained with Toto-3 (blue). (O–T) Zonal restriction of the tet-P2 allele driven by OMP-IRES-tTa examined by two-color RNA in situ hybridization. Coronal sections through olfactory epithelia of P90 OMP-IRES-tTa/tet-P2 animals were hybridized with RNA probes directed against GFP (green) (O and R), and against OMP (red) (P and S), in zonal region II/III (O–Q) and zonal region IV (R–T). Red and green channels are shown merged (Q and T). Nuclei were counterstained with Toto-3 (blue). (U–Z) Increase in frequency of expression of the tet-P2 allele over time. Coronal sections corresponding to zone II/III of the olfactory epithelia of OMP-IRES-tTa/tet-P2 animals subject to IHC with immunoserum directed against GFP (green) at P14 (U), P18 (V), P30 (W), P60 (X), P120 (Y), and P360 (Z). Nuclei counterstained with Toto-3 (blue).

Analyses of the patterns of transcription of OR genes has revealed that individual OR genes are expressed in neurons found in broad zones running across the neuroepithelium [6],[7],[33]; the expression of ORs is spatially restricted. The presence of such patterns of OR choice may reflect the positive effect of spatially restricted trans-acting factors that activate OR genes in a zonal manner. Alternatively, a common regulatory machinery may be at work across the neuroepithelium, with spatial restriction arising from the repression of OR genes outside of their zones. We have used the tet-modified P2 alleles to examine the phenomenon of zonal restriction of ORs, analyzing expression of tet-P2 and tet-P2Δ driven by tTa across the zones of the olfactory epithelium. A schematic of the olfactory epithelium depicting the P2 zone (shaded region) is shown in Figure 2A. In coronal sections of control P2-IRES-GFP lines (at P14) subject to IHC for GFP, the expression of P2 is characteristically restricted to the zone II/III region (Figure 2B and 2D), with the wild-type frequency of P2 expression in this zone ∼7% (Figure 2D). Neurons choosing the P2 receptor outside of its zone are rarely observed [10]. Despite the uniform presence of tTa across the neuroepithelium, we observed a zonal restriction of tTa-driven tet-P2 expression in coronal sections of OMP-IRES-tTa/tet-P2 mice at P14 (Figure 2C and 2E), similar to the pattern of wild-type P2 expression. Compared to wild-type controls (Figure 2B and 2D), the frequency of tTa-driven tet-P2 expression in the P2 zone is elevated to ∼14% of the cells (Figure 2C and 2E, and data not shown).

Analysis of epithelia from both tet-P2 and tet-P2Δ animals revealed a graded frequency of tTa-driven tet-P2 allele expression, despite the uniform and pervasive presence of the activating tTa transcription factor. Coronal sections of epithelia of adult CaMKII-tTa/tet-P2 mice examined in different zones (boxed areas shown in Figure 2A) reveal a frequency of choice of 74% from within the P2 zone (Figure 2G), 22% from a more dorsal position (Figure 2F), and 10% from the indicated ventral zone (Figure 2H). Analogous results were obtained for the tet-P2Δ allele, which lacks the P2 coding region. We observed tet-P2Δ expression in 60% of the neurons in the P2 zone (Figure 2J), in 16% of more dorsal neurons (Figure 2I), and in 10% of neurons in the ventral region (Figure 2K). In control experiments, we observed that the tet-M71 transgene, which is pervasively activated by OMP-IRES-tTa [18], is similarly activated across all zones in the olfactory epithelium by tTa expressed from the CaMKII-tTa transgene (Figure 2L–2N). This result confirms the uniform expression across the epithelium of tTa from the CaMKII transgene and suggests that the zonal restriction of tet-P2 expression is not a consequence of the limited availability of tTa. Further, we observed an analogous zonal restriction of tet-P2 when tTa expression was driven by OMP-IRES-tTa. In coronal sections subject to two-color RNA in situ hybridization with riboprobe for OMP and GFP transcripts, pervasive expression of OMP is observed from both the P2 zone (II/III) and the more ventral zone IV (Figure 2P and 2S). However, GFP RNA from the tet-P2 allele is observed in 80% of the cells in the P2 zone (Figure 2O) and in only 15% of the neurons in the more ventral region (Figure 2R). This zonal restriction of activation of the tet-P2 allele is specific for the tet operator inserted into the P2 locus, and has not been observed for tet-operator-driven OR transgenes, nor non-OR-containing transgenes [18],[19],[31]. The permissiveness of the tet-P2 allele is thus graded across the zones of OR expression in the olfactory epithelium, with the most frequent expression observed from within the wild-type P2 zone. Taken together, these results suggest that the spatial restriction observed for OR gene expression may be due to increasing levels of repression of the OR locus away from its zone (see Discussion).

In addition to the spatial regulation imposed on the tet-P2 allele, we observed a temporal change in the frequency of activation by tTa in the epithelia of OMP-IRES-tTa/tet-P2 mice. We examined coronal sections of OMP-IRES-tTa/tet-P2 mice at different ages (Figure 2U–2Z) and observed an increase in the frequency of expression of tet-P2, within the P2 zone, from 11% to approximately 96% over time. These data suggest a kinetic component to the activation of the locus, in which tTa may activate tet-P2 in an increasing proportion of the cells over time. Taken together these results imply that the zonal expression observed in OR regulation is due to graded repressive effects and that the OR coding region is not required for this spatial repression. These data support a model in which zonal control of receptor expression is mediated by repression of the OR promoter and depict a scenario in which the transcriptional permissiveness of the OR locus could dictate its frequency of choice.

### Pre-Activation of the tet-P2 Locus Alters Expression Frequency

**Figure 3:**
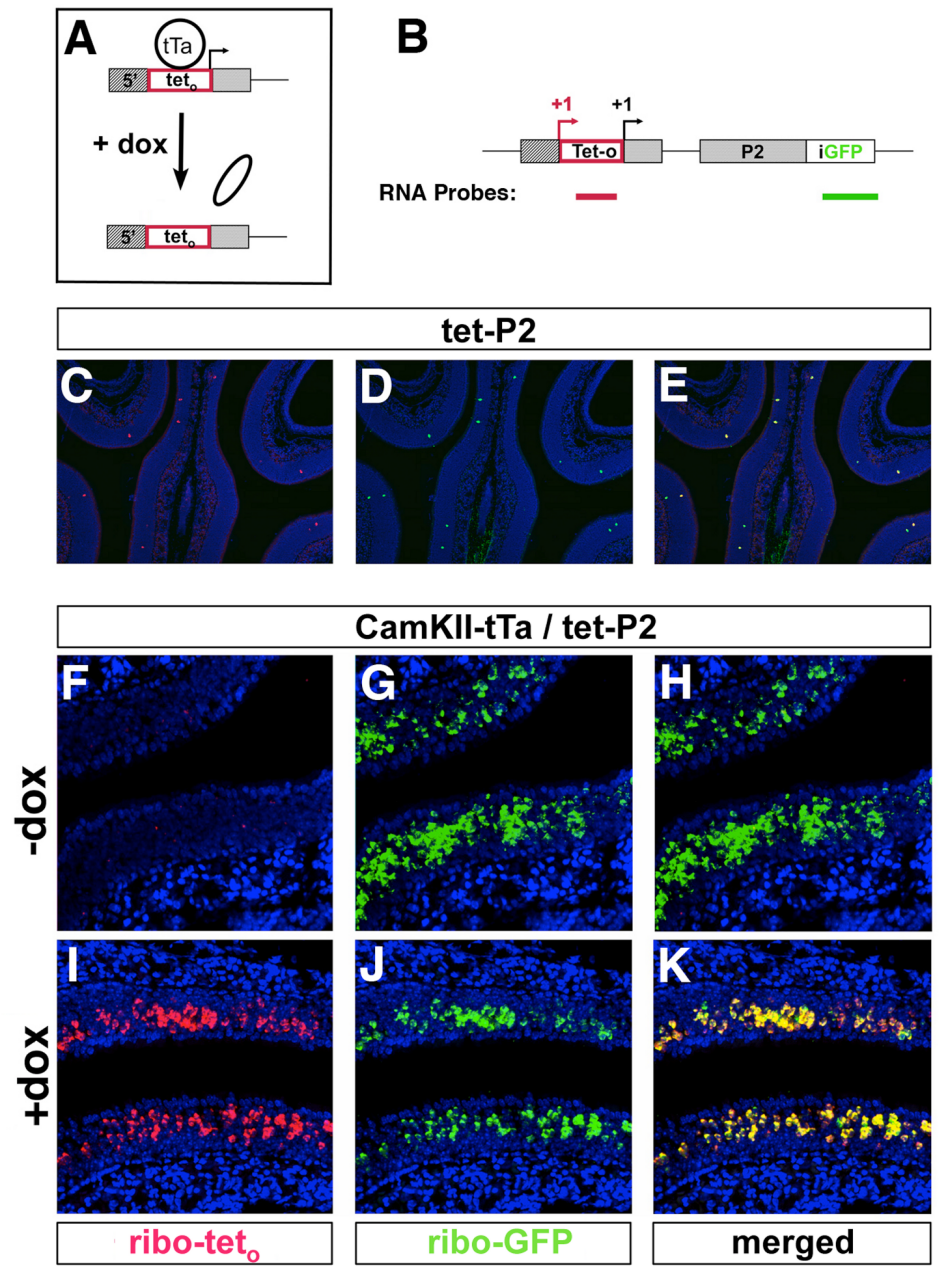
Pre-activation of tet-P2 leads to persistent expression independent of tTa. (A) Diagram of pre-activation strategy of tet-P2 with tTa by administration of doxycycline (dox). The tet-P2 locus is subject to activation by tTa until P60 by CaMKII-tTa. Doxycycline is administered, to ablate tTa binding, for 48 h prior to expression analysis by RNA in situ hybridization. (B) Diagram of the tet-P2 allele showing the location of RNA probes used to differentiate between wild-type (red “+1”) and tet_o_ (black “+1”) start sites of transcription. The RNA probe shown in red is derived from tet_o_ sequences and detects message initiated by the endogenous P2 promoter, while the probe shown in green is derived from GFP sequences and hybridizes to messages initiated from either endogenous P2 or tet_o_ promoters. (C–E) Control experiments demonstrate expression of the tet-P2 gene initiated from the wild-type P2 promoter. Coronal sections of a tet-P2 animal subject to RNA in situ hybridization with probe directed against the tet_o_ sequences (red) (C), with probe directed against GFP sequences (green) (D), and with red and green channels merged (E). Nuclei were counterstained with Toto-3 (blue). (F–H) Expression of the tet-P2 allele driven by CaMKII-tTa without doxycycline treatment at P60. Coronal sections of a tet-P2 CaMKII-tTa animal subject to RNA in situ hybridization with probe directed against the tet_o_ sequences (red) (F), with probe directed against GFP sequences (green) (G), and with red and green channels merged (H). Nuclei were counterstained by Toto-3 (blue). (I–K) Continuation of expression of the tet-P2 allele driven by CaMKII-tTa after 48 h of doxycycline treatment at P60. Coronal sections of a tet-P2 CaMKII-tTa animal subject to RNA in situ hybridization with probe directed against the tet_o_ sequences (red) (I), with probe directed against GFP sequences (green) (J), and with red and green channels merged (K). Nuclei were counterstained with Toto-3 (blue).

The apparent repressed state of OR chromatin, revealed through our functional in vivo studies of the tet-P2 allele (Figure 2) and described biochemically elsewhere [27], suggests that the OR selection mechanism could utilize the permissiveness of the OR locus to control the frequency of OR choice. In this scenario, we reasoned that pre-activation of the tet-P2 locus may alter its frequency of choice by the endogenous selection machinery by increasing the permissiveness of the locus. To test this possibility, we pre-activated the tet-P2 locus with tTa, then followed with a doxycycline treatment, which ablates tTa binding and thus transcriptional activation of the locus [28]. This approach is feasible because the tet-P2 allele retains its endogenous control sequences, moved 5′ by the insertion of the tet operator (Figure 1A) yet still functional. We can detect the use of the endogenous promoter (versus the tet operator) by monitoring the use of the endogenous transcriptional start site, upstream in the tet-P2 allele, using RNA in situ hybridization (Figure 3B). If the endogenous promoter is active, the tet-P2 transcript will contain both GFP (Figure 3B, green probe) and tet operator sequences (Figure 3B, red probe).

In initial experiments we performed two-color RNA in situ hybridization on coronal sections of olfactory epithelium from tet-P2 mice, with RNA probes for tet operator and GFP sequences. Neurons expressing the tet-P2 allele are identified by riboprobes for GFP (Figure 3D). Consistent with transcription directed by the endogenous promoter (and initiated upstream of the tet operator), these neurons are also detected by riboprobes for tet operator sequence (Figure 3C and 3E). In the absence of doxycycline, CaMKII-tTa drives massive over-expression of the tet-P2 allele, as demonstrated by RNA in situ hybridization with riboprobes for GFP (Figure 3G and 3H). However, we did not observe the use of the endogenous start site, upstream of the tet operator, unlike in the transcription of the tet-P2 allele on its own. This is demonstrated by the absence of RNA in situ signal generated by the tet operator probe (Figure 3F and 3H, within the P2 zone) or outside of the P2 zone (data not shown). These data are consistent with transcription initiation being directed by the tTa bound to the tet operator. Significantly, after 48 h of doxycycline treatment in mice bearing CaMKII-tTa and tet-P2, we observed that transcription of the tet-P2 allele persists at an over-expressed frequency in the P2 zone (Figure 3J and 3K), as well as outside this zone (data not shown). Concomitant with the persistent expression of tet-P2 is a switch in start site usage from the tet operator start site to the start site associated with the endogenous P2 promoter, as indicated by the detection of tet operator sequence included in the tet-P2 transcript (Figure 3I and 3K). The continued expression of the tet-P2 allele in the absence of tTa binding, coupled with the switch to the upstream transcriptional start site, suggests that the endogenous P2 promoter is active to direct transcription of the OR in doxycycline-treated mice.

These observations suggest that after an initial activation of the tet-P2 allele by tTa the P2 promoter is active on the gene. Further, these experiments imply that the frequency with which the endogenous expression machinery may assemble on an OR gene may be altered by the prior transcriptional state of that gene, which may influence the chromatin state of the locus. These data are consistent with a model in which the frequency of selection of an OR allele is proportional to the transcriptional permissiveness of its locus.

### A Developmental Change in OR Gene Permissiveness

**Figure 4:**
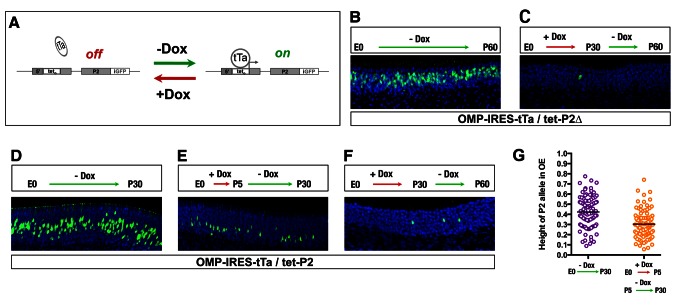
Developmental change in the permissiveness of the tet_o_-modified P2 alleles revealed by timed administration of doxycycline. (A) Diagram depicting strategy used in staged doxycycline administration experiments. (B) Coronal section through the olfactory epithelium of an OMP-IRES-tTa/tet-P2Δ animal maintained on food without doxycycline from E0 to P60. Expression of the tet-P2Δ allele is revealed by IHC with anti-GFP antiserum (green). Nuclei in all panels are counterstained with Toto-3 (blue). (C) Coronal section through the olfactory epithelium of an OMP-IRES-tTa/tet-P2Δ animal maintained on food containing doxycycline from E0 to P30 and then switched to undrugged food from P30 to P60. Expression of the tet-P2Δ allele is revealed by IHC with anti-GFP antiserum (green). (D) Coronal section through the olfactory epithelium of an OMP-IRES-tTa/tet-P2 animal maintained on food without doxycycline from E0 to P30. Expression of the tet-P2 allele is revealed by IHC with anti-GFP antiserum (green). (E) Coronal section through the olfactory epithelium of an OMP-IRES-tTa/tet-P2 animal maintained on food containing doxycycline from E0 to P5 and then switched to undrugged food from P5 to P30. Expression of the tet-P2 allele is revealed by IHC with anti-GFP antiserum (green). (F) Coronal section through the olfactory epithelium of an OMP-IRES-tTa/tet-P2 animal maintained on food containing doxycycline from E0 to P30 and then switched to undrugged food from P30 to P60. Expression of the tet-P2 allele is revealed by IHC with anti-GFP antiserum (green). (G) Distribution of tet-P2+ cells in the olfactory epithelium (OE) of OMP-IRES-tTa/tet-P2 animals maintained on food without doxycycline from E0 to P30 (purple) or maintained on food containing doxycycline from E0 to P5 and then switched to undrugged food from P5 to P30 (orange). The mean relative position, normalized to the height of the epithelium, of tet-P2+ cells from animals maintained on food without doxycycline or containing doxycycline was 0.424 and 0.304, respectively (*n* = 100, *p*<0.0001, unpaired *t*-test, two-tailed).

Once chosen, the expression of a functional OR may elicit a signal that feeds back and terminates the selection process, to maintain singular receptor choice in the neuron [15]–[17]. The mechanism by which the feedback process suppresses the expression of additional OR genes is unknown, but one model proposes that feedback induces a generalized repression of nonselected receptor alleles, making them inaccessible to the selection machinery [14]. To examine this stage of the OR selection process, we used conditional control of transcription of the tet-P2 alleles through doxycycline ablation of tTa binding [28]. By using staged administration of doxycycline we sought to determine changes in the transcriptional permissiveness of the tet-P2 locus at different times during the expression of the OR repertoire (Figure 4A). Using this approach we examined the permissiveness of the tet-P2 and tet-P2Δ alleles to tTa-mediated activation during different windows of time during development.

In control experiments, olfactory epithelia from OMP-IRES-tTa/tet-P2 mice were examined at P30 for tet-P2 expression, as revealed by detection of GFP. We observed tet-P2-expressing neurons in the P2 zone at frequencies consistent with previous analyses (Figures 4D and 2U, and data not shown). We next administered doxycycline-infused food to OMP-IRES-tTa/tet-P2 mice from embryonic day 0 (E0) to P5, via maternal feeding, and then maintained the animals without doxycycline to P30. In these mice we observed a dramatic decrease in the number of cells expressing tet-P2 and a basal shift in their distribution in the neuroepithelium (Figure 4E). To quantify this distribution we analyzed the relative position of tet-P2 cells in the olfactory epithelium. In olfactory epithelia of OMP-IRES-tTa/tet-P2 animals maintained without doxycycline, the mean relative position of tet-P2+ cells (normalized to the height of the epithelium) was 0.424 (Figure 4G, purple), whereas in olfactory epithelia of OMP-IRES-tTa/tet-P2 animals maintained on doxycycline from E0 to P5, and then released from doxycycline treatment from P5 to P30, the mean relative position of tet-P2+ cells was 0.304 (Figure 4G, orange, *p*<0.0001).

In a final doxycycline administration regimen, OMP-IRES-tTa/tet-P2 animals were fed doxycycline from E0 to P30, a point in time at which the majority of olfactory sensory neurons have chosen an OR to express [34], and then discontinued doxycycline-mediated inhibition of tTa from P30 to P60. In these mice we observed virtually no induction of the tet-P2 allele by tTa above that observed in the absence of tTa (Figure 4F). Taken together, these experiments provide evidence for a developmental change in the permissiveness of the locus to transcription directed by the tet operator and tTa concomitant with the development of the olfactory epithelium. This repression may be the result of the feedback signal elicited by functional ORs and could provide a mechanism for the maintenance of OR expression.

Previous studies have suggested that the sequence of the OR coding region itself plays a prominent, cis-acting role in feedback suppression of OR genes [19]. We therefore examined the transcriptional permissiveness of the tet-P2Δ allele, in which the coding region of P2 has been deleted, at a point in time at which the tet-P2 allele no longer allows tTa-directed transcription of the locus (Figure 4F). In control experiments the OMP-IRES-tTa/tet-P2Δ line was examined for tTa-driven expression of the tet-P2Δ allele by IHC at P60. Consistent with previous results, we observed high-frequency activation of the allele in the olfactory epithelium within the P2 zone (Figure 4B). However, when fed doxycycline from E0 to P30 and then analyzed after 30 additional days in the absence of doxycycline, we observed a dramatic decrease in the ability of tTa to induce expression of the tetP2Δ allele (Figure 4C), comparable to the level of suppression observed in analogous experiments with tet-P2 (Figure 4F). The developmental repression of the modified P2 alleles is specific to the P2 locus and was not observed for other tTa-driven transgenes in the olfactory epithelium. Importantly, when the M71-tg transgenic line was treated with doxycycline from E0 to P30 and analyzed after an additional 30 days in the absence of doxycycline, expression of the transgene was robustly induced (data not shown).

These results strongly suggest that unselected OR loci undergo developmental repression as olfactory sensory neurons mature and choose an OR. These data also demonstrate that this change in permissiveness of the locus does not require the participation of the OR coding region sequences. Together these data suggest that the OR promoter sequences are the sole mediators of this level of regulation. These data further suggest that there is a developmental window, terminating soon after the onset of OMP expression, during which OR loci are relatively permissive and after which they become highly repressed (see Discussion).

### Pervasive Expression of an OR Transgene Suppresses tet-P2 Alleles

The developmental change in the transcriptional permissiveness of the tet-P2 locus suggests a mechanism of feedback control of OR choice mediated by the OR promoter elements and effected through repression. To further examine this process we asked whether the tet-P2 allele would be subject to the suppressive effects of a ubiquitously expressed OR transgene (M71-tg) that we previously described [18]. This line carries an M71 transgenic construct, driven by tet_o_/tTa, that expresses the OR M71 and the marker protein tau-lacZ in greater than 95% of the olfactory sensory neurons. The pervasive expression of M71 suppresses the endogenous OR repertoire [18]. If M71-tg were to similarly suppress tet-P2, it would do so despite the continued presence of tTa. This would suggest a causal link between the change in transcriptional permissiveness observed at the P2 locus, and the feedback suppression exerted by the expression of the M71 transgene. We therefore crossed OMP-IRES-tTa/tet-P2 lines into mice bearing the M71 transgene and analyzed the tTa-driven expression of both tet_o_-linked loci.

**Figure 5:**
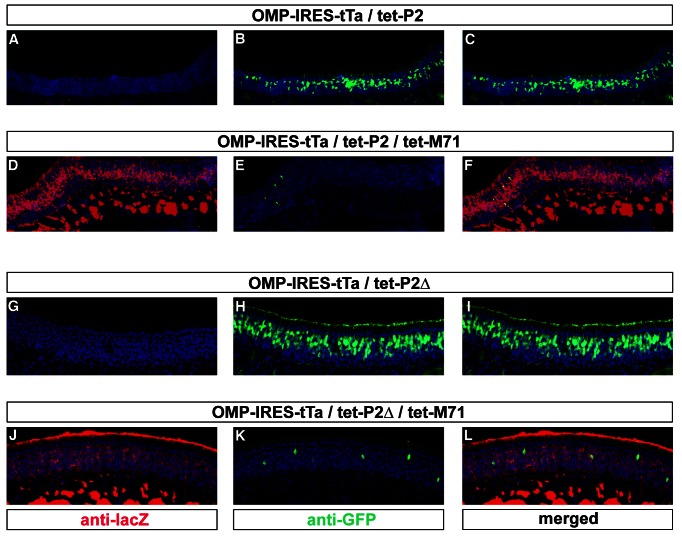
Suppression of tet_o_-modified P2 alleles by the pervasive expression of an OR transgene. (A–C) Coronal sections through the olfactory epithelium of an OMP-IRES-tTa/tet-P2 mouse subject to immunohistochemical detection of lacZ (red) (A) and GFP (green) (B), and with merged signals (C). Nuclei (blue) revealed by Toto-3 counterstaining. (D–F) Coronal sections through the olfactory epithelium of an OMP-IRES-tTa/tet-P2/tet-M71 animal subject to immunohistochemical detection of lacZ (red) (D) and GFP (green) (E), and with merged signals (F). Nuclei (blue) revealed by Toto-3 counterstaining. (G–I) Coronal sections through the olfactory epithelium of an OMP-IRES-tTa/tet-P2Δ mouse subject to immunohistochemical detection of lacZ (red) (G) and GFP (green) (H), and with merged signals (I). Nuclei (blue) revealed by Toto-3 counterstaining. (J–L) Coronal sections through the olfactory epithelium of an OMP-IRES-tTa/tet-P2Δ/tet-M71 mouse subject to immunohistochemical detection of lacZ (red) (J) and GFP (green) (K), and with merged signals (L). Nuclei (blue) revealed by Toto-3 counterstaining.

Coronal sections through the olfactory epithelia of OMP-IRES-tTa/M71-tg mice subject to immunohistochemical detection of lacZ reveal the pervasive expression of the M71 transgene (Figure 5D). Coronal sections through the olfactory epithelia of OMP-IRES-tTa/tet-P2 mice subject to immunohistochemical detection of GFP reveal typical frequencies of tTa-driven tet-P2 expression in the P2 zone at P30 (Figure 5B and 5C). In the OMP-IRES-tTa/tet-P2/M71-tg neuroepithelia, the M71 transgene is pervasively expressed (Figure 5D and 5F), while the expression of tet-P2 is markedly reduced (Figure 5E and 5F). We extended this experiment to the tet-P2Δ allele, observing typical frequencies of tTa-driven tet-P2Δ expression in the OMP-IRES-tTa/tet-P2Δ line (Figure 5H), while the expression of the M71 transgene similarly suppressed the tet-P2Δ allele (Figure 5J–5L) in a manner similar to that observed for tet-P2. The suppression of the tet-P2 loci is not due to competition for limited amounts of tTa, as we observed that the expression of other tet_o_-driven alleles remained unaffected by tet-M71 expression [18].

Thus tet_o_-linked P2 alleles are subject to the suppressive effects elicited by the pervasive expression of the M71 transgene similarly observed for the endogenous OR repertoire. The M71-transgene-mediated suppression occurs despite the continued presence of tTa and is observed at the tet-P2 locus even in the absence of the P2 coding region, further supporting a model of feedback suppression mediated by OR control elements rather than the OR open reading frame. It is interesting to note that the tet-P2 allele fails to suppress the M71 transgene, which implicates the cis-acting elements present in the endogenous OR locus that are absent from the M71 transgene in the feedback process (see Discussion).

### Limited Allelic Inclusion of tet-P2 Alleles

The expression of mammalian OR genes is monogenic, whereby only one member of the gene family is selected per cell, and monoallelic, with only one of the two copies of the gene transcribed [2],[5]. Monoallelic expression of ORs is not the result of an absolute inactivation of one of the two alleles, as lineage-marking studies have demonstrated their successive activation, known as “switching” [17]. OR genes are asymmetrically copied during S phase, with one allele duplicated early and one late [5]. It is possible that this staggered replication timing reflects differential epigenetic marking, biasing the likelihood of expression of one allele over the other. To explore the possibility of a functional nonequivalence between OR alleles, and to extend our analysis of the permissiveness of OR loci, we next asked whether tTa could drive biallelic OR expression in homozygous tet-P2 animals.

**Figure 6:**
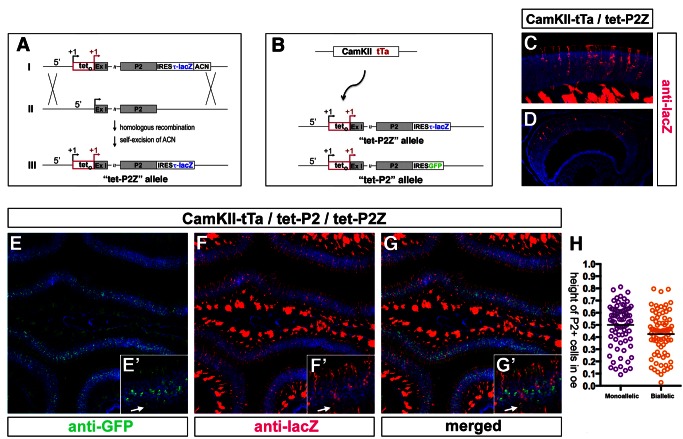
Construction of the tet-P2Z allele and predominant allelic exclusion of the homozygous tet_o_-modified P2 alleles. (A) Modification of the endogenous P2 locus by homologous recombination to generate the tet-P2Z allele. (I) The tet-P2Z targeting construct allows bicistronic expression of the P2 OR protein and the marker protein tau-lacZ, both driven by the tet operator inserted at the start site of transcription of the P2 locus. Flanking P2 promoter regions are preserved in the construct, shifted 5′ of the tet operator. (II) The unmodified genomic P2 locus. (III) Homologous recombination in mouse ES cells followed by self-excision of the ACN selection cassette yields the tet-P2Z allele. (B) Diagram of the genetic strategy used to for biallelic expression of the tet_o_-modified P2 alleles in the mouse olfactory epithelium in vivo. The tet-P2 and tet-P2Z alleles have the potential to be transcribed in all olfactory sensory neurons of the olfactory epithelium by the ubiquitous expression of tTa from the CaMKII-tTa transgene. (C and D) Expression of the tet-P2Z allele in the olfactory epithelium (C) and the VNO (D) revealed by IHC in coronal sections with antibody directed against lacZ (red) in a CaMKII-tTa/tet-P2Z animal. Nuclei are revealed by Toto-3 counterstain. (E–G) Expression of the tet-P2 and tet-P2Z alleles in a compound heterozygous animal CaMKII-tTa/tet-P2/tet-P2Z shown by immunohistochemical detection of GFP (green) (E) and lacZ (red) (F), and with merged signals (G). Nuclei are revealed by Toto-3 counterstaining. (E′–G′) High-power magnification of a region of the fields shown in panels (E–G), respectively. An olfactory neuron exhibiting biallelic expression of the tet-P2 alleles is shown by the arrows. (H) Distribution of single (purple) and double (orange) tet-P2+ cells in olfactory epithelia of CaMKII-tTa/tet-P2/tet-P2Z animals. The mean relative position, normalized to the height of the epithelium, of single tet-P2+ cells was 0.501 and of double tet-P2+ cells was 0.424 (*n* = 100, *p*<0.0068, unpaired *t*-test, two-tailed).

To conduct this experiment we constructed an additional tet-P2 allele in which the fusion protein tau-lacZ was used as a marker to allow us to distinguish expression of each of the two tet-P2 alleles (Figure 6B). We generated a genetically modified mouse line (tet-P2Z), by homologous recombination in mouse ES cells, identical to the tet-P2 line except that the fusion marker protein tau-lacZ, linked to an IRES, was inserted into the 3′ noncoding region of the P2 gene (Figure 6A). Thus, all neurons that express the tet-P2 allele would synthesize a bicistronic mRNA allowing the translation of both the P2 receptor and tau-lacZ proteins. Similarly to the GFP-marked tet-P2 allele, tet-P2Z expression driven by tTa was observed in comparable numbers of neurons in the main olfactory epithelium and the VNO (Figure 6C and 6D).

To examine the possibility of biallelic OR expression, we generated a mouse line carrying the CaMKII-tTa transgene and compound heterozygous for the tet-P2 modification: one allele marked with GFP and the other with tau-lacZ (Figure 6B). We analyzed expression of the tet-P2 alleles by immunohistochemical detection of GFP and lacZ in coronal sections of the olfactory epithelium of CaMKII-tTa/tet-P2/tet-P2Z animals. Both tet-P2 alleles were expressed in the epithelium at roughly equal frequencies, yet, remarkably, despite the genetic potential to express both, we observed that the vast majority of olfactory sensory neurons transcribed only one of the two tet-P2 alleles, with biallelic expression observed in only ∼3% of the neurons (Figure 6E–6G and 6E′–6G′). We further analyzed these data by measuring the height of double (tet-P2+tet-P2Z+) and single positive (tet-P2+ or tet-P2Z+) neurons in the epithelium and observed a difference between the two populations: double positive neurons were found lower in the epithelium than the single positives, with mean relative positions (normalized to the height of the epithelium) of 0.424 and 0.501, respectively (Figure 6H, *p* = 0.0068).

These results indicate that biallelic expression of an OR gene from its endogenous locus is possible but very infrequent, and may indicate a functional nonequivalence between the alleles. In this scenario an asymmetry exists between the two P2 alleles in which one has an increased likelihood of being activated over the other: the first allele activated would trigger the feedback process, repressing the other allele, which would lose the ability to be activated by tTa. Intriguingly, the distribution of cells expressing tet-P2 from both alleles is skewed basally, suggesting that allelic inclusion more often occurs in the younger sensory neurons (see Discussion).

## Discussion

The monogenic and monoallelic selection of ORs is a paradigmatic example of transcriptional selectivity whose underlying mechanism is not well understood. Conceptually, the process may be divided into an initiation phase, followed by a maintenance phase, during which the chosen OR allele is stably and exclusively expressed [14]. Initiation of OR expression likely involves a limiting process, and work from several labs has revealed that the maintenance phase is initiated by a feedback signal elicited by functional ORs [15]–[17].

We used a genetic strategy to examine OR selection in mice in which we inserted the tet_o_, an exogenous, conditional, non-olfactory promoter, into an OR locus in situ, by homologous integration. In this approach we made the assumption that the regulatory constraints imposed upon the endogenous OR promoter will similarly impinge upon the exogenous tet operator. This approach may then reveal parameters of the endogenous constraints on the OR locus in vivo, during initiation and maintenance, through the ability to toggle the activation of the tet operator on or off by doxycycline. The use of the tet operator thus allowed a functional “interrogation” of the OR locus, through which we revealed multiple axes of regulation of OR selection.

### Zonal Expression by Repression

One of the hallmarks of OR regulation is the localization of neurons expressing a given OR to a diffuse but restricted region, or zone, across the olfactory epithelium [6],[7],[33]. We have observed that the expression of the tet-P2 allele is similarly restricted, with the highest frequency of activation seen within the endogenous P2 zone, and diminishing frequency away from this region. We have demonstrated the pervasive expression of the tTa protein throughout the epithelium, and the restricted transcription of the tet-P2 gene. While it is a possibility that a positive-acting factor, localized to the P2 zone and acting in concert with the pervasively expressed tTa, could defeat repression at the OR locus in a zonal fashion, the most parsimonious explanation for this observation is that the OR gene is repressed outside of its zone. Further, the zonal repression observed for the tet-P2 allele does not possess sharp boundaries, but rather appears to exist as a gradient. In this scenario an OR zone may thus correspond to a local minimum of repression for the OR gene, and it is possible that a continuous gradient of chromatin states exists across the olfactory epithelium, such that OR genes exhibit a region of maximal permissiveness wherein they are most likely to be activated. In this model, each OR may have its own unique micro-zone, a scenario that would aid in the distribution of the OR repertoire across the epithelium and that is consistent with detailed analyses of OR zonal expression [33].

### Permissiveness of the OR Locus

Activation of the tet-P2 allele in olfactory sensory neurons by tTa is initially sparse, despite the pervasive expression of tTa across the epithelium (driven either by OMP or CaMKII). We observed that the frequency of expression of tet-P2 increases slowly over time, a phenomenology of expression that is in contrast to that seen for the M71 transgene, whose robust frequency of expression matches that of the tTa that drives it [18]. This finding immediately suggests that the P2 locus imposes a constraint on the tet operator that lowers the probability of its expression. The integration of this probability over time accounts then for the gradual increase in the appearance of tet-P2-positive neurons in the neuroepithelium. The probability of expression is not uniform across the epithelium, but rather has a maximum within the observed zone of the receptor and tapers off outside of this region.

This view of the initiation of OR selection is analogous to the “accessibility hypothesis” invoked to explain the regulation of V(D)J recombination [35],[36], and the limited permissiveness of the P2 locus we observed could provide a mechanism by which the initiation of OR expression could be tuned to a level where only one OR may be expressed in a given window of time. We have observed that the tet-P2 allele may continue to be over-expressed in the olfactory epithelium after initial tTa activation, despite ablation of tTa binding to the tet operator by doxycycline treatment (Figure 3). The tTa-independent over-expression is highest from within the P2 zone and tapers off away from it, in proportion to the tTa-driven frequency. Intriguingly, concomitant with the tTa-independent over-expression, we also observed a switch in start site usage in the transcription of the tet-P2 allele, upon doxycycline treatment, from the +1 of the tet operator to the endogenous start site (retained in the construct). These data may indicate that the endogenous OR selection machinery assembles on the P2 promoter and takes over expression of the gene when tTa-driven transcription is stopped by doxycycline. It is possible then that the endogenous machinery assembles on the P2 promoter, at higher than wild-type frequency, due to a change in the accessibility of the tet-P2 locus generated by the activation of the tet operator by tTa. Such a phenomenon is believed to be operant in Igκ gene rearrangement, where germline transcription alters chromatin structure and facilitates access of the recombination machinery [35]. Together these data are consistent with a mechanism in which the permissiveness of the OR locus limits access to the transcriptional machinery, to dictate the frequency of initial OR choice.

Recent work from the Lomvardas lab [27] has revealed biochemical hallmarks of OR chromatin that are consonant with our functional studies. Magklara et al. found that OR chromatin is enriched in histone H3 lysine 9 trimethylation and histone H4 lysine 20 trimethylation, consistent with features of both facultative and constitutive heterochromatin [27]. Further, they found that OR chromatin is compacted in the olfactory epithelium, a finding consistent with the limited transcriptional permissiveness to activation by tTa that we observed for the tet-P2 allele. Thus, the biochemical basis for the limited permissiveness observed for the OR locus may be the result of heterochromatization of OR loci. Finally, a recent analysis of OR promoters reveals the enrichment of IKZF1 binding sites within 100 bp of the transcriptional start site, a finding that could explain the targeting of repressive machinery to OR chromatin [37].

### Maintenance by Repression

The selection of a single OR gene by the olfactory sensory neuron is maintained by a feedback signal generated by functional receptor. We used the conditional expression afforded by the tet_o_/tTa system, through the staged administration of doxycycline, to examine OR maintenance, and observed a developmental change in the permissiveness of the tet-P2 locus. Our experiments demonstrate that by P30, tTa is effectively unable to activate the tet-P2 allele, suggesting that the tet-P2 locus, which allows activation of tet-P2 early, becomes fully repressed. The timing of this change in permissiveness is consistent with the age at which the olfactory epithelium has mostly completed maturation and OR expression has reached a plateau [38]. This repression of the OR locus may be the functional consequence of the feedback mechanism [16],[17],[39]. Previous studies have reported that the activation of tet_o_ by tTa is inefficient in certain populations of neurons in the central nervous system of adult mice, and it has been proposed that tet_o_ undergoes nonspecific silencing [40]. However, we, and others, have observed highly efficient activation of multiple tet_o_-driven transgenes in olfactory sensory neurons [18],[19],[30],[31],[41]. Thus, we argue that the developmental repression of the tet-P2 alleles reflects specific, physiological changes in the OR chromatin state.

A developmental repression of OR transgene expression has been reported in experiments in which the tet operator was used to drive transcription of OR coding regions [19]. In this study, the onset of OMP expression in the olfactory epithelium appeared to mark the point after which the OR transgene became repressed, and the authors argued that the OR coding region itself was the cis-acting sequence necessary for this phenomenon. However, our previous studies similarly examining the expression of tet_o_-regulated OR transgenes showed no such repressive effect [18]. In the present experiments, we used homologous recombination to allow an examination of the transcriptional permissiveness of an OR gene in its endogenous locus, with all flanking DNA elements preserved. In this more defined genomic context, we observed an increase in repression of the locus over time (Figure 4). We also observed repression of the tet-P2 alleles in the context of the M71 transgene (Figure 5). Importantly, neither the change in permissiveness nor the sensitivity to suppression by M71-tg was dependent on the OR coding sequences, as we observed similar effects with the tet-P2Δ allele, which lacks the P2 coding region. Thus, it is highly likely that the cis-acting elements that govern repression reside in the flanking DNA, including regions that have previously been defined as necessary for transcription of an OR locus [42], and that are similarly required for P2 locus expression (unpublished data).

Our experiments using the staged administration and withdrawal of doxycycline reveal a developmental window during which OR loci retain the ability to be activated (Figure 4E, and analysis in Figure 4G). The end of this period is likely demarcated by the developmental stage shortly after the onset of OMP expression. This window is revealed in Figure 4E, where tet-P2 may be activated by tTa supplied by OMP-IRES-tTa in younger OMP+ neurons, but not in the older OMP+ cells that reside more apically in the epithelium. Interestingly, this window is analogous to the time period during which we have previously observed OR “switching” prior to stabilization of OR choice [17].

It is important to note that the olfactory epithelium continually regenerates and thus consists of a heterogeneous mix of neurons born at different times. The olfactory sensory neurons occupy positions in the epithelium corresponding roughly to age: a developmental stratification in which newly born neurons are located more basally and move up to more apical layers as they age. Maximal neurogenesis is observed in the first postnatal weeks and slows after a month to maintain the population of sensory neurons throughout the life of the animal. The activation of tet-P2 observed after doxycycline withdrawal therefore may occur either in cells that were OMP+ before withdrawal or those added to the OMP+ population after withdrawal. The marked inability of tTa to activate tet-P2 in older OMP+ neurons, when doxycycline is discontinued at P5, clearly shows that tet-P2 is repressed in this population. The subpopulation of cells that allows activation of tet-P2 by tTa in the lower OMP+ stratum may be composed of neurons previously resident in this layer, or added to it subsequent to the discontinuation of doxycycline treatment. In either scenario, it is clear that in the older neurons that are apical to this region, tet-P2 has lost the ability to be activated by tTa.

### The Problem of Monoallelism Revisited

Olfactory neurons choose one OR and express it randomly from one allele [5],[43]. Unlike the random and heritable inactivation of one of the two X chromosomes, both OR alleles can be activated in the same neuron, albeit sequentially, especially if the first allele chosen is nonfunctional [17]. We have examined the phenomenon of monoallelic OR expression using two tet-P2 alleles marked with two different reporter proteins (lacZ and GFP). Despite the genetic potential of cells to express both tet-P2 alleles, we observed biallelic expression only 3% of the time. As the overall frequency of expression of the tet-P2 allele is roughly 50%, we should expect to see both alleles transcribed in the epithelium 25% of the time. What could account for this discrepancy? It is possible that the low permissiveness to transcription of any OR allele is such that feedback repression may occur before any subsequent OR activation. It is also possible that a functional asymmetry exists between OR alleles, such that in a given cell, one allele is more likely to be activated than the other. The fact that receptor alleles display replication-timing asymmetry [5] suggests that a differential marking of alleles may exist and be used to stagger activation, providing enough time between possible selection events to allow feedback repression and ensure monoallelic expression.

The intriguing observation that cells expressing tet-P2 biallelically were found in a more basal region than those expressing tet-P2 monoallelically suggests that cells expressing tet-P2 biallelically make up a younger neuronal subpopulation. It is possible, therefore, that a refinement mechanism exists that prevents such biallelic expression, whereby the maturing neurons force the extinction of one of the two alleles. It is further possible that a competitive process between OR alleles underlies this mechanism of monoallelic expression.

### A Model of OR Selection

We thus favor a model of OR selection in which kinetic mechanisms ensure the initial stochastic selection of an OR allele. In this model, OR loci are in a semi-permissive state that limits initial OR activation to ensure that only one OR gene may be randomly activated within a given window of time. The inefficiency of this initial selection process ensures that the first functional receptor gene chosen will trigger the feedback mechanism prior to any subsequent OR activation. In this way, the initial expression of OR is probabilistic, resembling variegated activation [44]. The chosen OR allele is then maintained as the sole receptor, after the feedback mechanism triggers a change in the chromatin of the nonselected OR alleles, making them inaccessible.

By what mechanism would the selected OR allele remain transcriptionally active in the context of the feedback repression? It is possible that there is a unique nuclear compartment involved in the maintenance of OR choice that protects the selected allele. In this scenario, the single activated OR allele would gain entrance into this specialized compartment and be shielded from feedback repression, thus stably maintaining singular OR choice. The existence of such a compartment may be revealed by the observation that an olfactory locus control region, the H element, on Chromosome 14 [16],[24],[25] associates with active receptor alleles in trans [22]. This association may mark a specialized transcriptional factory [20] for OR expression required for the maintenance of singular OR expression.

## Materials and Methods

### Generation of Targeted P2 Alleles

#### tet-P2-GFP and tet-P2-tau-lacZ

A plasmid of the P2 OR gene-targeting construct [10] was modified by PCR to introduce a ClaI site at the transcriptional start (+1) of the P2 gene [21]. Into this ClaI site we introduced a fragment containing the hCMV minimal promoter with seven repeats of the tet operator located just upstream, which was derived from plasmid pUHD 10-3 by PCR [28]. This construct was further modified by the insertion of a cassette containing an IRES element followed by either GFP (tet-P2-GFP) or the fusion protein tau-lacZ (tet-P2-tau-lacZ), as well as the self-excising angiotensin-converting-enzyme–Cre-LoxP-Neo^R^-LoxP (ACN) construct [45], just 3′ to the stop codon of the P2 gene. These constructs were linearized and electroporated into 129/SvEv ES cells. Homologous recombinants were identified by Southern analyses using a 3′ external probe [10], as well as a 5′ internal probe derived from the tet operator. Targeted clones were injected into C57BL/6 blastocysts to produce chimeras that transmitted the modified P2 alleles through their germlines.

#### tet-P2Δ-GFP

A plasmid of the P2 OR deletion gene-targeting construct [10] was modified by PCR to introduce a ClaI site at the transcriptional start (+1) of the P2 gene. Into this ClaI site we introduced a fragment containing the hCMV minimal promoter with seven repeats of the tet operator located just upstream, which was derived from plasmid pUHD 10-3 by PCR. This construct was further modified by the insertion of a cassette containing an IRES element followed by GFP, as well as the self-excising ACN construct, into a PacI site generated at the site of the deletion of the P2 coding region. The construct was linearized and electroporated into 129/SvEv ES cells. Homologous recombinants were identified by Southern analyses using a 3′ external probe as well as a 5′ internal probe. One targeted clone was injected into C57BL/6 blastocysts to produce chimeras that transmitted the modified allele through their germlines.

### Immunohistochemistry and Quantitation

Olfactory turbinates were dissected out and immediately fixed in freshly prepared 1% paraformaldehyde (Electron Microscopy Sciences) in 1× PBS on ice for 60 min, followed by decalcification in 0.45 M EDTA, 1× PBS, for 18 h at 4°C. Tissue was frozen in O.C.T. compound (Sakura-Fintek), and 16-µm sections were cut on a cryo-microtome (Leica) and collected on Superfrost Plus slides (Fisher Scientific). IHC was performed with rabbit antisera against GFP (Molecular Probes) used at 1∶1,000, goat antiserum directed against beta-galactosidase (Biogenesis) used at 1∶1,000, and rabbit anti-VP16 (Abcam) used at 1∶500. Secondary antibodies (donkey) conjugated to Cy3 (Jackson Labs Technologies) or Alexa 488 (Molecular Probes) were used to visualize primary antisera in conjunction with Toto-3 nuclear counterstain (Molecular Probes). Stained sections were visualized, and whole-mount visualization of endogenous GFP fluorescence was performed, with Zeiss 510 and 710 laser-scanning confocal microscopes.

Relative cell position in the olfactory epithelium was determined by measuring the distance of the receptor positive neuron from the neuroepithelium–lamina propria interface divided by the basal-to-apical height of the epithelium. Graph points represents an individual cell, with *n* = 100 for each genotype. All measurements were performed using Image J software, and graphs were created, and corresponding statistics performed, using GraphPad Prism 6.0 software.

### RNA In Situ Hybridization

Olfactory turbinates were dissected out and immediately fixed in freshly prepared 1% paraformaldehyde (Electron Microscopy Sciences) in 1× PBS on ice for 60 min, followed by decalcification in 0.45 M EDTA, 1× PBS, for 18 h at 4°C. Tissue was frozen in O.C.T. compound (Sakura-Fintek), and 16-µm sections were cut on a cryo-microtome (Leica) and collected on Superfrost Plus slides (Fisher Scientific). Two-color RNA in situ hybridizations were performed using riboprobes labeled with either digoxigenin (dig) or fluorescein isothiocyanate (FITC) derivatized ribonucleotides (Roche) by either T7 or SP6 RNA polymerase (Promega). Probes were hybridized [17] on the sections for 18 h at 68°C in hybridization buffer containing 50% formamide (Sigma). Probes labeled with dig were detected by sheep anti-dig conjugated to horseradish peroxidase (Roche), and visualized using Cy3 tyramide (PerkinElmer) following manufacturer's instructions. FITC-labeled probes were detected by sheep anti-FITC horseradish peroxidase following inactivation of the anti-dig horseradish peroxidase with 0.05% sodium azide in TNB buffer (TSA Kit, PerkinElmer), and visualized with FITC tyramide (PerkinElmer). Nuclei were counterstained with Toto-3, 1∶1,000 (Molecular Probes). Slides were visualized with Zeiss 510 and 710 laser-scanning confocal microscopes.

### Doxycycline Feeding Experiments

Conditional expression of the tet-P2 alleles was accomplished by treatment with doxycycline, which ablates the binding of tTa to tet_o_ in the operator element. Mice were fed doxycycline-infused food (Bio-Serv Dox diet, 200 mg/kg) from E0, through maternal feeding, to postnatal ages indicated, to accomplish staged activation or deactivation of the tet-P2 allele.

## Abbreviations

ACN: angiotensin-converting-enzyme–Cre-LoxP-Neo^R^-LoxP
dig: digoxigenin
E0: embryonic day 0
ES cell: embryonic stem cell
FITC: fluorescein isothiocyanate
IHC: immunohistochemistry
IRES: internal ribosomal entry site
OR: odorant receptor
P[number]: postnatal day [number]
tet_o_: tetracycline-dependent transactivator responsive promoter
tTa: tetracycline-dependent transactivator
VNO: vomeronasal organ
